# Effect of a *ctrA* promoter mutation, causing a reduction in CtrA abundance, on the cell cycle and development of *Caulobacter crescentus*

**DOI:** 10.1186/1471-2180-13-166

**Published:** 2013-07-18

**Authors:** Patrick D Curtis, David Klein, Yves V Brun

**Affiliations:** 1 Department of Biology, University of Mississippi, 402 Shoemaker, University, MS 38677, USA; 2 Department of Biology, Indiana University, 1001 E. 3rd St., Bloomington, IN 47405, USA

**Keywords:** CtrA, P2 promoter, PodJ, Pleiotrophic

## Abstract

**Background:**

Polar development during the alphaproteobacterium *Caulobacter crescentus* cell cycle is integrated to the point that individual mutations can have pleiotropic effects on the synthesis of polar organelles. Disruption of the genes encoding the histidine kinase PleC, or its localization factor PodJ, disrupts synthesis or functionality of pili, flagella and adhesive holdfast. However, the mechanism by which these mutations affect polar development is not well understood. The aim of this study was to identify new regulators that control multiple aspects of polar organelle development.

**Results:**

To identify mutants with pleiotropic polar organelle synthesis defects, transposon mutagenesis was performed and mutants were selected based resistance to the pili-tropic bacteriophage ΦCbK. Mutants were then screened for defects in motility and holdfast production. Only a single *podJ*/*pleC*-independent mutant was isolated which had defects in all three phenotypes. Directed phage assays confirmed the phage resistance phenotype, while the strain demonstrated a similar dispersal radius as a *podJ* mutant in swarm agar, and treatment with a fluorescent lectin that labels the holdfast showed no staining for this mutant. The transposon had inserted into the promoter region of *ctrA*, a gene encoding a master transcriptional regulator of the cell cycle, disrupting native transcription but still allowing reduced transcriptional activity and protein production of this essential protein. Transcriptional fusions showed that essential genes controlled by CtrA exhibited minor to moderate changes in expression in the *ctrA* promoter mutant, while the *pilA* gene, encoding the subunit of the pilus filament, had a drastic decrease in gene expression. Introduction of a plasmid-born copy of *ctrA* under its native promoter complemented the phage resistance and holdfast defects, as well as a moderate cell morphology defect, but not the swarming defect.

**Conclusions:**

A mutation was identified that caused pleiotropic defects in polar organelle synthesis, and revealed the surprising result that some CtrA-dependent promoters are more sensitive to changes in CtrA concentration than others. However, the fact that no pleiotropic mutations were found in new regulators suggests that downstream signaling of PleC/PodJ is either essential, redundant, or branching such that all three phenotypes were not simultaneously affected.

## Background

The best-studied asymmetrically dividing prokaryote is the alphaproteobacterium *Caulobacter crescentus*. At each cell division, predivisional cells of *C*. *crescentus* localize different structures at the cell poles: a single flagellum occupies the pole that will be inherited by the swarmer cell and pili are synthesized at this pole after division, whereas a narrow extension of the cell envelope (the stalk) tipped by an adhesive structure (the holdfast) occupies the opposite pole that will give rise to the stalked cell. The stalked cell is able to restart the cell cycle immediately after division, whereas the swarmer cell is unable to initiate DNA replication until it differentiates into a stalked cell.

The *C*. *crescentus* cell cycle and developmental program are controlled by three master regulators: CtrA, GcrA, and DnaA (for review, see [1]). These proteins are regulated such that each one reaches maximal abundance during a different stage of the cell cycle. DnaA reaches peak abundance at initiation of DNA replication occurring in stalked cells, GcrA peaks after DNA replication in early predivisional cells, and CtrA peaks in late predivisional and swarmer stages [2]. All three proteins are required for regulating transcription of different suites of genes. DnaA activates genes involved in chromosome partitioning, nucleotide biosynthesis, and DNA replication, recombination and repair [3], and initiates replication of the chromosome. DnaA is also required for transcription of *gcrA*[3]. GcrA activates transcription of genes involved in DNA replication, recombination and repair different from DnaA targets [3-5]. GcrA also activates genes required for polar development (including *pleC* and *podJ*, both of which are also activated by DnaA [3,4]). CtrA, in turn, regulates at least 95 genes in 55 operons: some are repressed (for example *gcrA* and *podJ*[4,6]) whereas others are activated (such as the pilin subunit gene *pilA*, flagellum synthesis cascade initiation, and the holdfast anchor operon [7]). Additionally, CtrA binds to the chromosome at the origin of replication where it represses the initiation of DNA replication [8]. Furthermore, CtrA both activates and represses its own promoters.

The *ctrA* gene has two promoters: P1 and P2 [9]. The weaker upstream P1 promoter is activated first. P1 activation requires that the promoter be in the hemi-methylated state, meaning that DNA replication has initiated and the replication fork has passed the P1 promoter. The P1 promoter is also directly activated by GcrA [4,9,10]. The low level of expression from the GcrA-activated *ctrA* P1 promoter allows some CtrA protein to accumulate. Once sufficient CtrA has accumulated, it represses the P1 promoter (as well as *gcrA* expression) and activates the strong downstream P2 promoter [9], leading to a burst of CtrA production and activity.

The sequential activation of the master regulators forms the timeline by which developmental processes are regulated and coordinated. In particular, GcrA contributes to the expression of the key developmental regulators, the histidine kinase PleC and the polar localization factor PodJ. Loss of either protein causes pleiotropic defects in development. A *pleC* mutant does not synthesize a stalk, holdfast or pili, and though the flagellum is made, flagellar rotation is not activated and the flagellum is not shed during the swarmer cell differentiation [11-13]. A *podJ* mutant, like *pleC*, does not synthesize holdfast or pili or shed its flagellum, but it does synthesize a stalk and activates its flagellum, however its motility is impaired in low-percentage agar as compared to wild type [6,14,15].

To further elucidate the pathways that lead to these pleiotropic phenotypes a genetic approach was used. We conducted a transposon mutagenesis screen, selecting for resistance to phage ΦCbK, which requires pili for infection, and screening for defects in motility and adhesion, which require the flagellum and holdfast respectively. In this work we report the identification of a transposon insertion in the promoter region of *ctrA* that causes a drastic reduction of CtrA accumulation, resulting in pleiotropic phenotypes bearing similarities to the *pleC* and *podJ* phenotypes.

## Results and discussion

### A transposon mutation causes a pleiotropic phenotype

*C*. *crescentus* wild-type strain CB15 was mutagenized with the *mariner* transposon and mutants resistant to the bacteriophage ΦCbK were isolated to enrich for mutants defective in pilus synthesis. Once isolated, mutants were microscopically examined to identify defects in the presence or placement of stalks, the formation of rosettes (groups of cells adhered to each other by their holdfasts), or in swimming motility. Strains exhibiting a defect in any of these features were further analyzed for motility defects on swarm plates. A total of 330 Kan^R^ ΦCbK^R^ mutants were screened and classified into 7 categories (A-G) based on these polar phenotypes (Table 1). The majority of mutants (297) were morphologically indistinguishable from wild-type when grown in PYE liquid media (Class A), suggesting that they were pili synthesis mutants; these were not analyzed further. Classes B, C and D had stalks, formed rosettes, and differed from each other only in their swarming phenotype, ranging from no swarming (Class B) to the formation of small swarms (Class C) and finally to moderate-sized swarms resembling those of a *podJ* mutant (Class D). Class E exhibited phenotypes identical to a *podJ* mutant (stalks, no rosettes and moderate swarming), and all were confirmed by Southern analysis to have insertions in *podJ*. Class F resembled the known *pleC* phenotype (stalkless, no rosettes, no swarming), and all mutants in this class were shown to have insertions in *pleC*.

**Table 1:** **Classes of ΦCbK**-**resistant mutants isolated**

	**# of mutants**	**Stalks**^**a**^	**Rosettes**^**a**^	**Swimming**^**a**^	**Swarming**^**b**^
Wild-type	Control	+	+	+	++++
Δ*podJ*	Control	+	-	+	++
Δ*pleC*	Control	-	-	-	+
Class A	297	+	+	+	ND
Class B	5	+	+	-	-
Class C	3	+	+	-	+
Class D	3	+	+	-	++
Class E (*podJ*)	8	+	-	+	++
Class F (*pleC*)	13	+/−	-	+	+
Class G (YB3558)	1	+/−	+/−	+	+++

^a^Determined by visual identification in liquid culture. ^b^Determined by assaying motility of cells through low-percentage agar. Phenotypes scored on a relative scale from fully motile (++++) to non-motile (−). ND = not determined.

**Figure 1:**
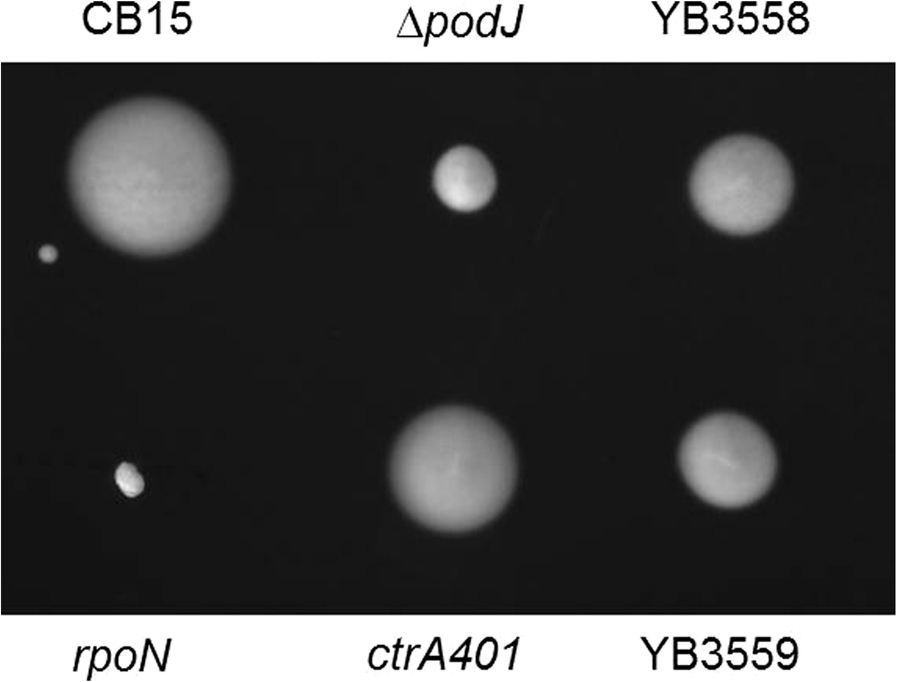
**Motility of YB3558, YB3559 and ctrA401.** The swarm assay was performed as described in the Methods. The non-motile *rpoN* mutant and the polar development mutant Δ*podJ* are included. YB3558 displays an intermediate swarming defect, between Δ*podJ* and wild-type levels, similar to a *ctrA* temperature sensitive lethal allele grown under permissive conditions. Complementation of YB3558 with a wild-type *ctrA* gene under native control (YB3559) does not restore the swarming phenotype.

**Figure 2:**
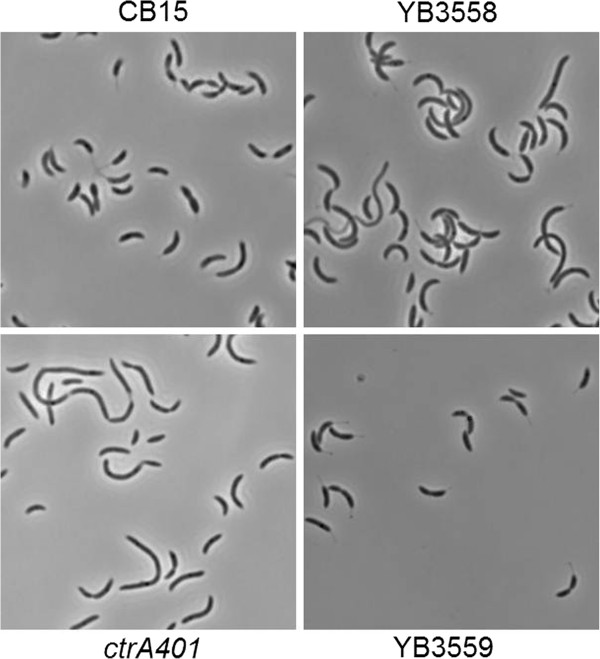
**Morphology of YB3558 and YB3559.** Phase micrographs of exponentially growing cultures of CB15, YB3558, *ctrA401* and YB3559 at 30°C. YB3558 displays increased filamentation, increased percentage of cells with multiple constrictions, and a stalkless phenotype. These defects are also seen in the *ctrA* temperature sensitive strain grown under permissive conditions. Complementation of YB3558 with a wild-type *ctrA* gene under native control (YB3559) restores cell morphology to that of wild-type.

**Figure 3:**
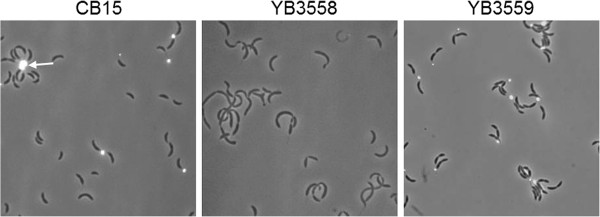
**Lectin binding of wild-type CB15, YB3558 and YB3559.** Wild-type, YB3558 and YB3559 cells were treated with FITC-conjugated WGA lectin and imaged as described in the Methods. WGA lectin binds to holdfast and is seen as a fluorescent focus when imaged (example indicated with white arrow, WT panel). Wild-type cells display holdfast at the tips of stalks, while the YB3558 mutant produces no holdfast. Complementation of YB3558 with a *ctrA* gene under native control (YB3559) restores holdfast synthesis.

One mutant, M134 and later the transduced derivative YB3558, did not fit into any of the other classes. Similar to *podJ* mutants, this mutant produces moderate sized swarms (Figure 1), yet the morphology of the cells was variable and did not resemble *podJ* mutant cells which exhibit normal morphology. Analysis of the cell morphology of YB3558 revealed that it had numerous deficiencies as compared to wild-type CB15 (Figures 2 and 3). Cells displayed a moderate filamentation phenotype. A cell division defect was apparent in an increased percentage of cells with at least one visible constriction. In CB15 predivisional cells comprised 17% of the total population, whereas in YB3558, 35% of the population was had at least one constriction. Furthermore, the prevalence of cells with multiple constrictions was increased from less than 1% in CB15 to 3% of the total cell population (or ~10% of predivisional cells) in YB3558. More severe defects were observed in stalk synthesis (Figures 2 and 3). In CB15, 91% of predivisional cells had a visible stalk as compared to only 32% in YB3558. Staining of holdfast polysaccharide with FITC-WGA indicated that 8% of predivisional cells of YB3558 had a holdfast as compared to 74% of predivisional CB15 cells (Figure 3).

**Figure 4:**
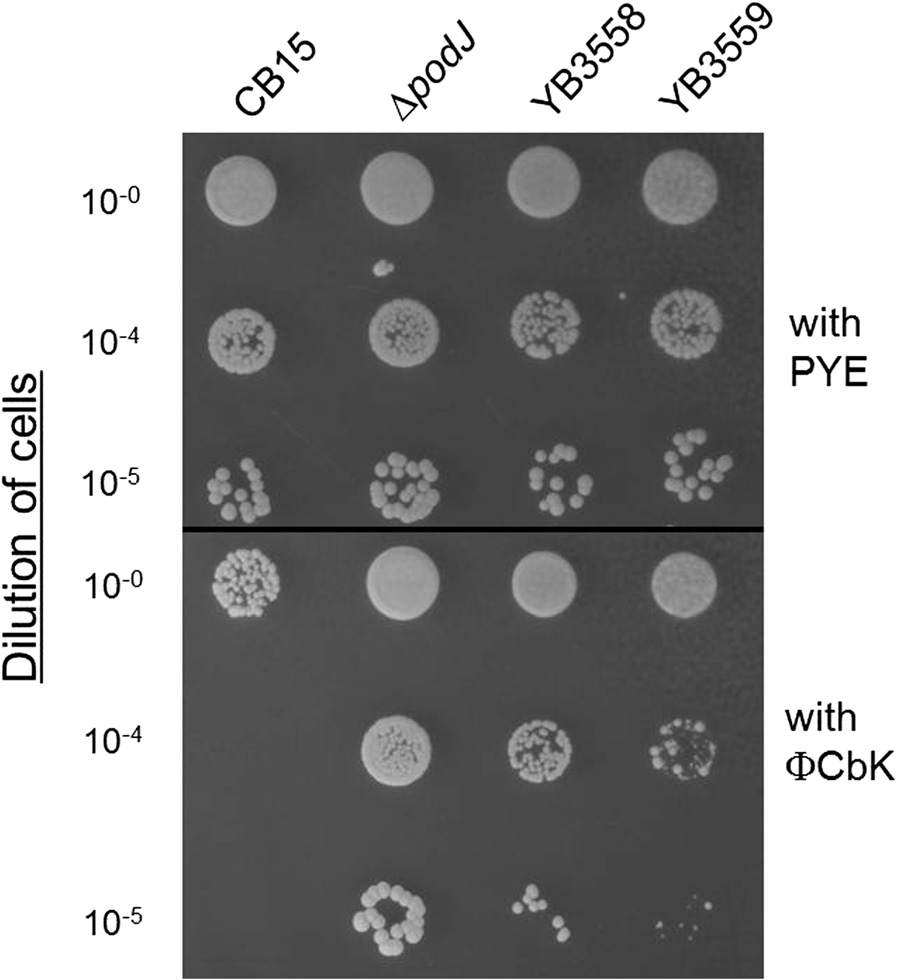
**Phage resistance of YB3558 and YB3559.** Resistance to the *Caulobacter* phage ΦCbK was assayed as described in the Methods. Dilutions of cells were mixed with phage stock and spotted onto PYE plates. CB15 is sensitive to the phage, Δ*podJ* is resistant, and YB3558 shows virtually the same phage resistance as Δ*podJ*. Complementation with *ctrA* under native control (YB3559) decreases phage resistance to nearly wild-type levels.

**Figure 5:**
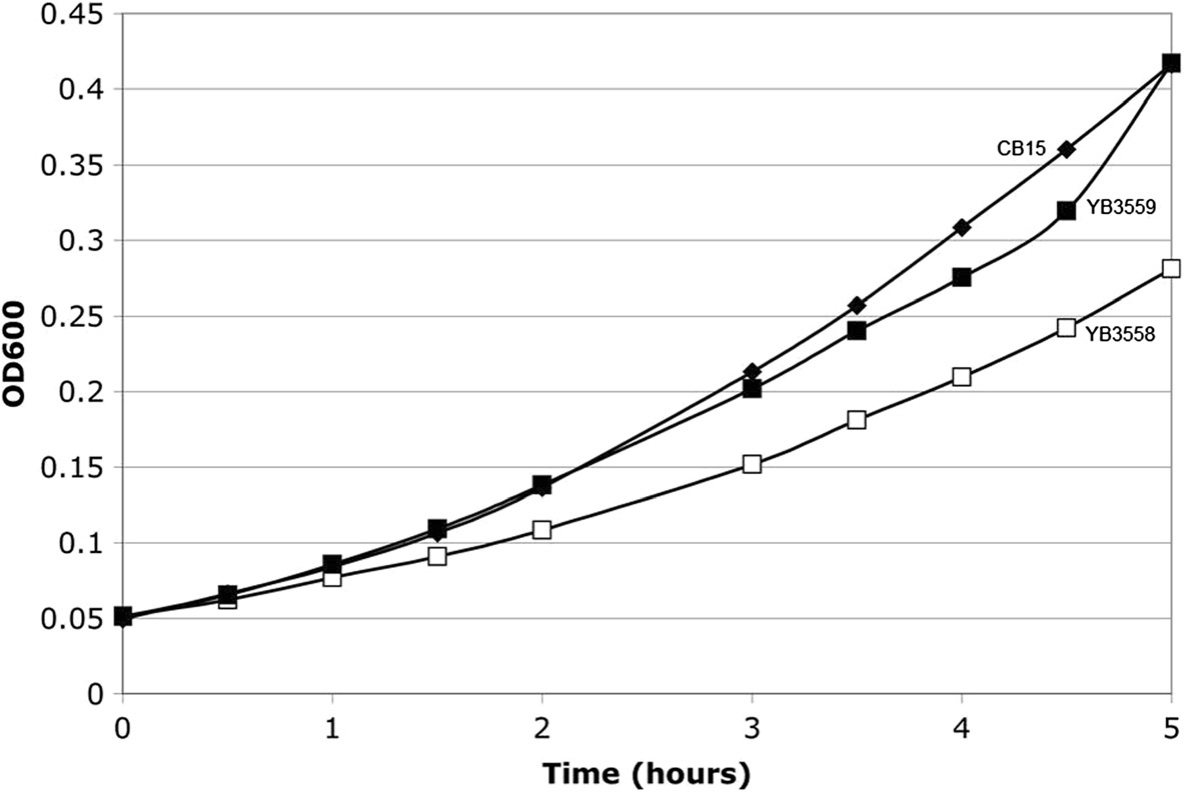
**Growth rate of YB3558 and YB3559.** Growth curves of CB15 (black diamonds), YB3558 (open squares) and YB3559 (black squares). YB3558 shows slower growth than wild-type. Complementation with *ctrA* under native control (YB3559) restores wild-type growth.

YB3558 demonstrated similar phage resistance to ΦCbK as *podJ* in a phage sensitivity assay. When serial dilutions of cells were mixed with phage stock and spotted on PYE plates (Figure 4), cell growth in lower dilutions was slightly less dense than that of the fully resistant *podJ* mutant. However, relative to wild-type cells, YB3558 exhibited significant phage resistance, allowing survival at the lower dilutions of phage. Finally, YB3558 grew more slowly than wild-type with a doubling time of 123 minutes as compared to 97 minutes (Figure 5).

### YB3558 transposon insertion is in the *ctrA* regulatory region

**Figure 6:**
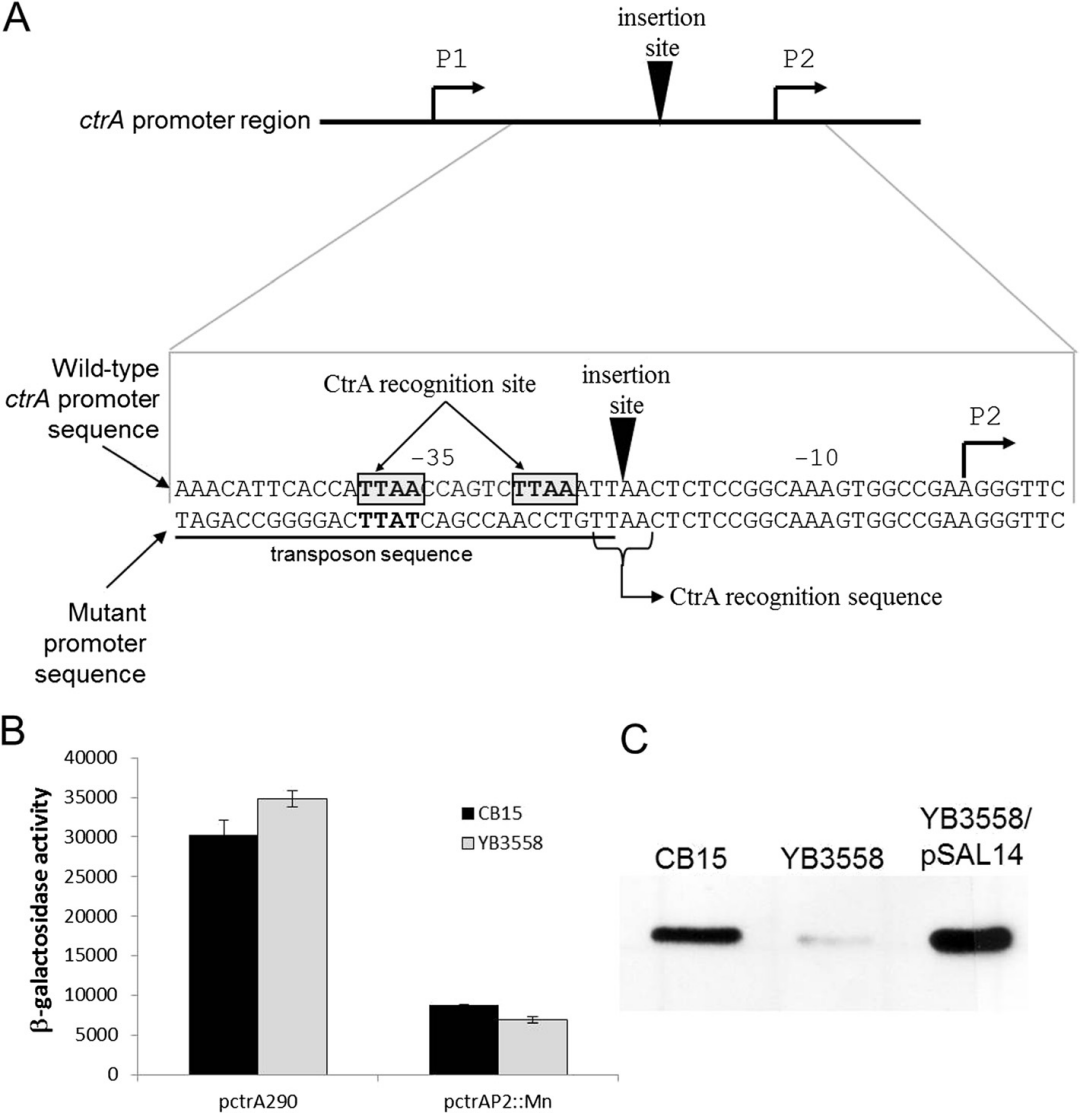
**Insertion site of the transposon in YB3558 and effect on CtrA abundance. A)** Location of the insertion site relative to the P1 and P2 promoters of *ctrA* and sequence of the wild-type *ctrA* P2 promoter and the mutated *ctrAP2*::*Mn* promoter. Shaded boxes indicate CtrA recognition sequence half sites. Transcription start site is indicated [9]. Triangle indicates site of transposon insertion. Transposon sequence is underlined. **B)** Expression of wild-type (pctrA290) and mutant (pctrAP2::Mn) *ctrA* promoters in wild-type and YB3558 strains. β-galactosidase assays were performed on exponentially growing cultures as described in the Methods (absorbance measurements for this experiment were carried out on a Nanodrop 2000 (Thermo Scientific) with a 0.1 mm light path and therefore activities are not Miller Units, and instead have been labeled β-galactosidase Activity). The mutant promoter displays a large reduction in activity compared to the wild-type promoter in both CB15 and YB3558. The wild-type promoter displays an expression level in YB3558 similar to that in CB15. **C)** Western Analysis of CtrA abundance in YB3558 and YB3559. Western blot analysis was conducted on an equal OD_600_ of each strain. Blots were probed with α-CtrA primary antibody and HRP-conjugated goat anti-rabbit secondary antibody (Biorad) and the signal detected with the Supersignal Pico substrate (Pierce). YB3558 demonstrates significantly reduced CtrA abundance, while complementation with *ctrA* under native control (YB3559) restores CtrA to near wild-type levels.

Mapping of the mutation in YB3558 indicated that the transposon had inserted between the −10 and −35 sites of the *ctrA* P2 promoter (Figure 6A) [9], indicating that the pleitropic defects observed are not due to disruption of a polar development gene like *podJ* or *pleC*, but alteration of one of the master regulators directly. The exact site of insertion and the sequence of the end of the transposon were such that the −35 site remained somewhat intact. Of the two TTAA half-sites required for CtrA-binding [9], one was slightly altered (TTAA→TTAT), and the other was completely abolished (Figure 6A). The half site that was completely abolished is very likely necessary for efficient transcription of CtrA-controlled promoters, including *ctrA* itself. While the end of the transposon creates another half site, it is separated by an additional 5 bases from the first half site. Previous mutational analysis of the consensus CtrA recognition sequence revealed that the drastic alteration of either TTAA half site in the recognition sequence TTAA-n7-TTAA greatly reduces transcription of the promoter, and alteration of the downstream TTAA half site can also abolish cell-cycle regulation [16]. Because YB3558 does not have the complete recognition site essential for efficient induction of the P2 promoter by CtrA, and the P1 promoter is separated from the translational start site by the full length of the transposon, we hypothesized that transcription of the *ctrA* gene is reduced in the YB3558 mutant, and the resultant reduction of CtrA protein could be the cause of the pleiotropic phenotypes observed in this strain.

To verify this hypothesis, we generated a fusion of the *ctrA* mutant promoter from YB3558 to *lacZ* and compared expression from this promoter to the wild-type *ctrA* promoter in both CB15 and YB3558 during exponential growth (Figure 6B). Expression from the mutant promoter was only 20% of wild-type *ctrA* promoter expression in YB3558 and 29% wild-type *ctrA* promoter expression in the wild-type strain indicating that even when CtrA is present and its activity is normal (as it is in CB15), the mutant promoter is not efficiently transcribed.

Since the mutant *ctrA* promoter (containing the transposon insertion) from YB3558 demonstrated reduced activity in wild-type, suggesting *ctrA* transcription is reduced in YB3558, Western blot analysis was performed to measure CtrA abundance. Results showed that CtrA is expressed at a much lower level in YB3558 than in CB15 (Figure 6C). Subsequent quantification of band intensities from six Western blots showed that CtrA is present at approximately 22 +/− 5% of the wild-type CB15 level, demonstrating that the reduced transcription resulting from the transposon insertion leads to drastically lower CtrA protein levels.

### Polar development defects are linked to altered CtrA abundance/activity

In order to determine if the lower CtrA levels are involved in the polar development defects found in YB3558, similar assays that were performed on YB3558 were also performed on *ctrA401*, a temperature sensitive CtrA allele [17]. At the restrictive temperature the allele is lethal, but at the permissive temperature *ctrA*-dependent promoters demonstrate altered transcription patterns that indicate that CtrA401 has impaired function. Phenotypic analysis demonstrates that a *ctrA401* mutant has a reduced swarming phenotype (Figure 1), as well as morphological defects (Figure 2), both of which mirror those of YB3558.

Plasmid pSAL14 was introduced into YB3558, creating strain YB3559. pSAL14 is a low copy plasmid carrying a copy of the *ctrA* gene with its native promoter [17]. Introduction of the plasmid restored CtrA production to slightly above wild-type levels (Figure 6C). Phenotypic analysis of YB3559 demonstrated that *ctrA* complementation restores cell morphology (Figure 2) and holdfast synthesis (Figure 3) to wild-type phenotypes, and growth rate to near wild-type levels (Figure 5). Phage sensitivity was increased over that of the parent YB3558 (Figure 4), but not complemented to full wild-type levels (it should be noted pinprick-sized colonies are likely spontaneous suppressors). Interestingly, *ctrA* complementation appears to have no effect on the swarming defect of YB3558 (Figure 1). The causal relationship between reduced CtrA abundance and the reduced swarming phenotype in this mutant is unknown.

### Effect of the *ctrA* promoter mutation on transcription of developmentally regulated genes

Though expression of the mutant *ctrA* promoter was reduced regardless of the strain harboring it (Figure 6B), the wild-type *ctrA* promoter displayed similar expression levels when placed in YB3558, indicating its activity is resistant to the severe reduction in CtrA protein levels in that strain. Given that CtrA is a global regulatory protein for both essential (e.g. cell division) and non-essential (e.g. polar development) genes, and that the drastic CtrA reduction in YB3558 leads to polar developmental defects but the strain is still viable, we hypothesized that transcription of CtrA-regulated genes essential for cell survival will be less affected by CtrA reduction in YB3558 than those that are essential for less important cellular functions. Thus we investigated the transcription level of several CtrA-regulated genes in CB15 and YB3558.

**Figure 7:**
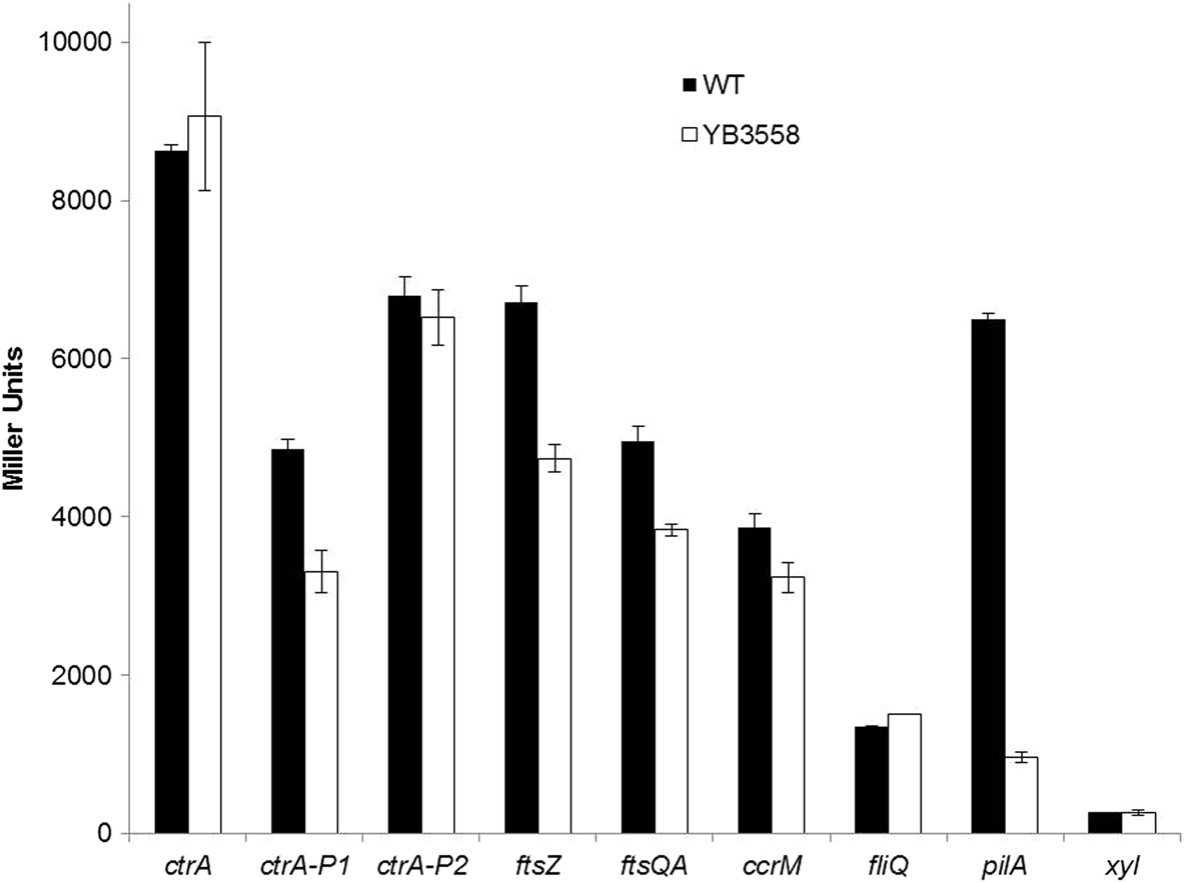
**Expression of CtrA**-**dependent promoters in wild**-**type and YB3558 strains.** β-galactosidase assays were performed on exponentially growing cultures as described in the Methods. CtrA-dependent promoters of essential cell process genes show little-to-no change between wild-type and YB3558, while the *pilA* promoter shows a drastic difference in expression between the strains.

Plasmids bearing transcriptional *lacZ* fusions were introduced into both wild type and YB3558 strains. The promoters for the reporter constructs were *ctrA* (pctrA290, [9]), *ctrA* P1 (pctrA-P1, [9]) *ctrA* P2 (pctrA-P2, [9]), *ftsZ* (plac290/HB2.0BP, [18]), *ftsQA* (pMSP8LC, [19]), *ccrM* (pCS148, [20]), *fliQ* (pWZ162, [21]) and *pilA* (pJS70, [22]) as well as *lacZ* under the control of a xylose inducible promoter to serve as a negative control (pCS225, [23]). Exponential phase cultures were assayed for β-galactosidase activity (Figure 7). Total transcriptional activity from the *ctrA* promoter was unaffected, though there was a reduction of activity from the weak P1 promoter, but not the stronger P2. Activity from these promoters is dependent upon many factors, one of them being CtrA protein abundance. It is possible that even though CtrA abundance in YB3558 is severely reduced, it is more than enough to activate the P2 promoter.

*ftsZ* and *ftsQA* promoters had a moderate reduction in activity, and the *ccrM* promoter had a slight reduction in activity. These genes are essential for viability. The moderate reduction in transcription for these genes agrees with the hypothesis that genes involved in essential cell cycle processes would not be severely affected by the reduction in CtrA in YB3558. In contrast, the *pilA* promoter exhibited a drastic decrease in activity, as would be expected given the selection by which this mutant was obtained. However, activity from the *fliQ* promoter (*fliQ* is a flagellar biosynthesis gene and not essential) was largely unaffected. It is not clear why this promoter is unaffected while the *pilA* promoter shows such a difference in activity. It could be that the *pilA* promoter is much more sensitive to CtrA levels.

Regulation of *pilA* is controlled not only by CtrA, but by SciP. SciP interacts with CtrA to prevent transcription of genes positively regulated by CtrA, such as *pilA*, in swarmer and late predivisional cells [24]. It is possible that the dramatic decrease in *pilA* promoter activity in YB3558 is not from CtrA abundance itself, but an indirect effect of reduced CtrA abundance leading to increased SciP activity. However, CtrA positively regulates transcription of *sciP* and the strong reduction of CtrA activity in the YB3558 mutant should lead to a decrease in SciP levels, not an increase. In agreement with this hypothesis it has been shown that a site-directed mutation that abolishes transcription from the *ctrA* P1 promoter caused a strong reduction of CtrA abundance [25], similar to that of the YB3558 mutation in this study, and this lead to significantly reduced expression of SciP, down to 19% of wild-type level [25]. The *ctrA* P1 mutant also had morphological and growth defects similar to those found here, and several assays demonstrated that CcrM transcription and translation was largely unaffected, agreeing with our results. Therefore it is unlikely that the effects observed on gene expression are the result of increased SciP activity.

Some CtrA-dependent promoters appear more resilient to changes in CtrA concentration than others. It has been shown previously that promoters that deviate from the canonical TTAA-N7-TTAA CtrA binding site have a lower CtrA binding affinity [26,27]. It is possible promoters that are more susceptible to changes in CtrA concentration have more divergent CtrA binding sites, causing them to have lower CtrA affinity and thus lower binding site occupancy at lower CtrA concentrations such as found in YB3558. A list of CtrA binding sites from each of the transcriptional fusions used in Figure 7 (excluding the *xyl* control) is shown in Table 2. The CtrA binding region for each gene was determined experimentally by DNA footprinting (see references in Table 2). The *ctrA*-P2, *ccrM* and *fliQ* reporters displayed the least change in YB3558 compared to wild-type, indicating expression from these promoters is more resilient to changes in CtrA concentration. The *ctrA*-P2 site is well characterized as TTAA-N6-TTAA with an additional TTAA half site 1 bp downstream. This binding site is relatively close to the canonical structure. The binding sites for *ccrM* and *fliQ* are TTAA-N7-CTAA and CTAA-N7-TTAA respectively. Each binding site differs from the canonical structure by a single base pair substitution. Therefore, the promoters displaying little change in YB3558 all are relatively similar to the known CtrA binding sequence. The *ctrA*-P1, *ftsZ*, *pilA* and to a lesser extent *ftsQA* fusions all displayed noticeable changes in expression in YB3558 compared to wild-type. The *ctrA*-P1 binding site consists of a single TTAA half site and obviously diverges greatly from the consensus CtrA binding site. The *ftsQA* site is TTAA-N7-CTAA, the same as the *ccrM* binding site, though *ftsQA* only displays a moderate change in transcription inYB3558. However, the *ftsZ* promoter region, which shows the second largest transcriptional change in YB3558, has a perfectly canonical TTAA-N7-TTAA binding site. Therefore it is unlikely that varying promoter affinities due to divergence from the consensus CtrA binding site can fully explain the changes (or lack thereof) for CtrA-dependent promoters in YB3558, though they may still contribute.

**Table 2:** **CtrA binding sites for CtrA**-**regulated genes**

**Gene**	**CtrA binding site**	**Ref.**
Canonical CtrA	xxxx**TTAA**xxxxxxx**TTAA**xxx	[17]
*ctrA*-P1	ATTCGCAAATCAGA**TTAA**CCA	[9]
*ctrA*-P2	CCA**TTAA**CCAGTC**TTAA**A**TTAA**CTC	
*ftsZ*	CAG**TTAA**CCGCCGA**TTAA**CGA	[18]
*ftsQA*	CCG**TTAT**GACGACA**TTAA**CGA	[19]
*ccrM*	TGG**TTAA**CGGCCCG**CTAA**CCA	[26]
*fliQ*	CCC**CTAA**CGCCCTG**TTAA**CCA	[17]
*pilA*–Region 1	CTG**TTTA**CTGGCCA**TTAA**GTG	[22]
Region 2	TGG**TTAA**GAACAA**ATAA**CGG**TAAA**TACAAA**TAAA**CCA	
Region 3	TGG**TCAA**CAAAAGA**CTAA**AAT	

TTAA half sites are indicated in bold.

Though the genes used for analysis in this study mostly have single CtrA-binding sites close to the consensus, the *pilA* gene, which displays drastically reduced transcription in YB3558 compared to wild-type, appears different compared to the other genes presented in regards to CtrA regulation. CtrA was shown to the bind to three distinct regions in the *pilA* promoter area. Region 1 has a TTTA-N7-TTAA binding site straddling the −35 site. Region 2, 19 bp upstream of Region 1, has two potential CtrA binding sites, TTAA-N6-ATAA and TAAA-N6-TAAA, separated by 3 bp. Region 3, 71 bp upstream of Region 2, has a single TCAA-N7-CTAA binding site. Though the Region 1 binding site is relatively close to the consensus sequence, all the other binding sites diverge greatly from the consensus in sequence and/or half-site spacing. Clearly CtrA regulation of *pilA* is more complex than that of the other genes presented. Perhaps the divergent binding sites have low affinity for CtrA and the multiple weak binding sites create cooperative CtrA binding necessary to achieve maximal *pilA* expression. It would be plausible that this scenario (multiple weak sites working together) would be quite sensitive to changes in CtrA protein levels, leading to the drastic reduction in transcription seen in YB35587. Further analysis of CtrA regulation of *pilA* will prove informative.

Is it possible that promoters more susceptible to changes in CtrA concentration/activity account for all the pleiotropic defects observed in *podJ* and *pleC* strains? Current understanding of PleC’s role (and thus PodJ’s) in developmental signaling is to regulate phosphorylation levels of another signaling protein DivK, which in turn regulates the activity of the CckA phosphorelay that controls CtrA activation [28,29]. A *pleC* mutant should have reduced CtrA levels, similar to the CtrA phenotype found in this study. Though CtrA protein levels in *pleC* are similar to wild-type, there is a significant decrease in CtrA phosphorylation [30]. Also in agreement with this hypothesis, reduced CtrA levels have been implicated as contributing to the null-pili phenotype of *podJ* mutants [31]. However, the other polar development phenotypes are not as well explained by CtrA-promoter effects. The known link between CtrA and flagellar motility in *C*. *crescentus* is that CtrA initiates the flagellum synthesis cascade [20]. The *fliQ*-*lacZ* reporter demonstrates that the synthesis cascade is unaffected, which agrees with the fact that both *pleC* and *podJ* mutants produce flagella. CtrA must affect motility in a way other than synthesis of the flagellum, possibly two ways since the flagellum is paralyzed in a *pleC* mutant but capable of rotation in a *podJ* mutant. The effect of CtrA on motility appears to be independent of CtrA abundance as complementation of CtrA abundance by pSAL14 failed to restore wild-type motility to YB3558 (Figure 1). If the effect is not dependent on CtrA abundance, it may be dependent on timing of CtrA activity. Expression from the mutant promoter in YB3558 is likely constitutive, and may lead to early induction of whatever CtrA-dependent pathway is involved in motility other than flagellum synthesis. However, the CckA/ChpT pathway that controls CtrA activity should not be perturbed in this mutant, so even though CtrA could be produced constitutively, its activity should still be properly regulated. The full link between CtrA and motility is still a mystery.

The connection between CtrA and holdfast synthesis is also not clear. While it is known that at least some of the holdfast synthesis genes display changes in transcription activity during the cell cycle [32], and microarray experiments have shown that holdfast genes have altered transcription in a *ctrA* mutant [7,33], it has also been shown that holdfast synthesis can be stimulated in swarmer cells when they contact a surface [34], and that developmental holdfast synthesis is also likely regulated by cyclic-di-GMP levels [35]. We have recently shown that the holdfast synthesis and anchoring machineries are synthesized and polarly localized in predivisional cells in preparation for holdfast synthesis in the next cell cycle [36,37]. Therefore, it is likely that CtrA regulates the synthesis of the holdfast synthesis-anchoring machinery in predivisional cells, but that the activation of this machinery is regulated by surface contact and developmental signals. The additional possibility that CtrA abundance effects post-transcriptional regulation of holdfast synthesis cannot be ruled out. However, both effects on motility and post-transcriptional effects on holdfast synthesis could be downstream effects of CtrA-dependent decrease in promoter activity of one or more other regulators.

## Conclusions

In this study we performed a detailed mutagenesis selection/screen to identify new regulators that control multiple aspects of polar development similar to known developmental regulators PleC and PodJ. Our results suggest that potential regulators downstream of those already known may be essential, redundant or branched. In the process we found evidence that suggests at least some of the pleiotrophic phenotypes are the result different affinities between CtrA and CtrA-dependent promoters.

## Methods

### Media and growth conditions

All *C*. *crescentus* strains were grown at 30°C in peptone yeast extract (PYE) media [38]. When appropriate, kanamycin (5 μg/ml liquid, 20 μg/ml solid), chloramphenicol (0.5 μg/ml liquid or 1 μg/ml solid), tetracycline (1 μg/ml liquid or 2 μg/ml solid) and nalidixic acid (20 μg/ml) were used. *Escherichia coli* strains were grown at 37°C in Luria-Bertani (LB) medium [39] with kanamycin (50 μg/ml), chloramphenicol (20 μg/ml liquid or 30 μg/ml solid), ampicillin (50 μg/ml liquid or 100 μg/ml solid), or tetracycline (12 μg/ml liquid or 12 μg/ml solid).

### Transposon mutagenesis and selection of ΦCbK^R^ mutants

The plasmid pFD1 [40], carrying the *mariner* transposon and the transposase gene, was introduced into *C*. *crescentus* strain CB15 (wild-type) by conjugation with *E*. *coli* strain YB2028 (SM10λpir (pFD1)). Cells from five independent conjugations were pooled and frozen at -80°C. Aliquots of cells were thawed, mixed with undiluted *Caulobacter* phage ΦCbK stock (~10^10^ pfu/ml), plated on PYE supplemented with kanamycin and nalidixic acid and incubated at 30°C for several days until Kan^R^ ΦCbK^R^ colonies appeared.

### Screening mutants

#### Visual screening

Overnight cultures of all ΦCbK^R^ mutants were observed with a 100× objective on a Nikon Optiphot-2 microscope. Strains were qualitatively scored on three phenotypes: presence of rosettes, presence of stalks, and presence of motile swarmer cells.

#### Phage resistance

Strains were grown overnight, normalized to equal OD_600_ and diluted to 10^0^, 10^-4^ and 10^-5^. Cell dilutions were mixed in equal volumes with ΦCbK (~10^10^ pfu/ml) or plain PYE. The mixture was incubated at room temp for 10 minutes, then 5 μl spots were placed onto PYE plates. The plates were incubated at 30°C for 3–5 days. Relative resistance was determined by the number and size of colonies that appeared.

#### Confirmation of transposon mutant phenotypes and identification of genes

The kanamycin marker in strains of interest were transduced into *C*. *crescentus* strain CB15 with the phage ΦCr30, using a standard transduction protocol [41]. Kan^R^ colonies were isolated and overnight liquid cultures were shown to have the same phenotype as the parent strain.

Genomic DNA was isolated using a phenol/chloroform extraction method. Briefly, cells were grown overnight at 30°C in 3 ml PYE + kanamycin. The entire culture was pelleted by centrifugation, and resuspended in cold TE pH 7.5 to a final volume of 500 μl. Lysozyme (Sigma) and RNAse (Amresco) were added to final concentrations of 1 mg/ml and 0.1 mg/ml respectively, and the mixture was incubated at 37°C for 30 min before adding 0.1 volumes of 10% w/v SDS. Proteinase K (Amresco) was added to a final concentration of 1 mg/ml. The solution was mixed gently and incubated at 50°C for 2 hours with occasional mixing. 50 μl of sodium chloride (5 M) was added, and the DNA was extracted 3–4 times with one volume of phenol/chloroform/isoamyl alcohol (25:24:1), followed by two extractions with one volume of chloroform. After extraction, DNA was precipitated with 0.6 volumes of isopropanol, washed twice with 70% v/v ethanol, allowed to dry, and resuspended in 50 μl dH_2_O.

#### Southern blot analysis

In order to identify mutants with insertions in *podJ* and *pleC*, Southern blot analysis was used to analyze the positions of the *mariner* insertions in mutants with phenotypes similar to *podJ* and *pleC*. Probes were prepared with DIG-High Prime DNA Labeling and Detection Starter Kit I (Roche). A 2.1 kb *podJ* probe was PCR amplified from CB15 genomic DNA using primers 5podJ2508 and 3podJ4522 (Table 3) and probed against *Sfi*I-digested chromosomal DNA. A 2.9 kb *pleC* probe was PCR amplified from CB15 genomic DNA using primers pleCfor and pleCrev (Table 3) and probed against *Xho*I-digested chromosomal DNA.

**Table 3:** Primers used in this study

	
5podJ2508	GCCTGGTGGGCCGCTCTGAT
3podJ4522	CGGTTGGGGACATCGTCCCC
pleCfor	ATCGTCGTCGACTTGCCCGCGCCC
pleCrev	GCCAGCAAGGCGCTCGGCTGACGA
pBGST181	ATGGCAAGATCCTGGTAT
pBGST182	CGATAATGTCGGGCAATC
MarRseq	CGGGTATCGCTCTTGAAGGGA
M134UP	GGACGAGTCGGAATTCCAGACCG
M134DN	GCCTTCAGACTCTAGAATGAGTTCG
CtrAlacUp	CAGAACGCCGGAATTCCGTCCGTGA

For strains of interest that did not have insertions in *podJ* or *pleC*, genomic DNA (~3 μg) was digested with *Pst*I and separated on an agarose gel. DNA was excised from the gel area found to include the band seen by Southern analysis using a probe for the kanamycin resistance gene. The DNA was isolated from the gel using the Qiaquick Gel Extraction kit (Qiagen) and ligated to *Pst*I-digested pKSII+ (Stratagene) overnight at 16°C. The ligation was electroporated into *E*. *coli* strain DH5α (F’, ϕ80d*lacZ*ΔM15, Δ(*lacZYA*-*argF*)U169, *endA1*, *recA1*, *hsdR17* (rk-, mk+), *deoR*, *thi*-*1*, *supE44*, λ-, *gyrA96*, *relA1*). Amp^R^ Kan^R^ colonies were isolated, and plasmid DNA was purified.

#### DNA sequencing

Plasmids were sequenced with primer MarRseq (Table 3) using Big Dye version 3.1 (Applied Biosystems), and run on an ABI3730 DNA Analyzer at the Indiana Molecular Biology Institute (Indiana University). The transposon insertion site was identified in the sequence, and the gene was identified by a Basic Local Alignment Search Tool (BLAST) search against the *C*. *crescentus* genome (TIGR - http://blast.ncbi.nlm.nih.gov/Blast.cgi?PAGE_TYPE=BlastSearch&PROG_DEF=blastn&BLAST_PROG_DEF=megaBlast&BLAST_SPEC=MicrobialGenomes_155892&DB_GROUP=AllMG).

### Characterization of the YB3558 mutant

#### Visual analysis

Cultures of YB3558 were grown overnight in PYE with kanamycin, diluted to an OD_600_ of approximately 0.15, and allowed to grow to an OD_600_ of 0.5-0.6, then observed using 100X Plan Apo objective on a Nikon Eclipse E800 microscope. Images were captured using a Princeton Instruments 1317 cooled CCD camera and processed with Metamorph v. 4.5 (Universal Imaging Corporation). Staining of holdfast with fluorescein isothiocyanate-wheat germ agglutinin (FITC-WGA) was performed as described previously [32]. Fluorescence was observed on the Nikon E800 and images were processed using Metamorph.

#### Growth curves

Strains were grown overnight in PYE supplemented with appropriate antibiotics and diluted to an OD_600_ of 0.1 in fresh PYE with no antibiotic. They were allowed to grow for two doublings (to OD_600_ of ~0.4) and diluted again to an OD_600_ of 0.05 in 10 ml of PYE. 100 μl of the culture was removed and its OD_600_ recorded every 30 minutes for 5 hours.

#### Swarm assay

Strains were grown overnight in PYE supplemented with appropriate antibiotics, diluted to an OD_600_ of 0.1, and allowed to grow for two doublings (to OD_600_ of ~0.4). All strains were diluted to an equal OD_600_ and 1 μl of the culture was injected into a 0.3% Agar PYE plate. This was incubated at room temperature for 5–7 days in a humid container.

#### Complementation

Plasmid pSAL14 [17], carrying a wild-type copy of the *ctrA* gene, was transformed into YB3558. The resulting strain, YB3559, was assayed for complementation of the phenotypes seen in YB3558.

#### Western analysis

To examine levels of CtrA in mixed culture, exponentially growing cells were collected and resuspended to equal OD_600_ in a final volume of 100 μl in 1X SDS loading buffer (62.5 mM Tris–HCl pH 6.8, 10% v/v glycerol, 2% w/v SDS, 0.05% v/v β-mercaptoethanol, 0.0025% w/v Bromophenol blue). 15 μl of this sample was separated on a 10% SDS-polyacrylamide gel and transferred to a nitrocellulose membrane. The membrane was probed with α-CtrA serum [42] at 1:10,000 dilution. The membrane was then probed with HRP-conjugated goat anti-rabbit secondary antibody (Biorad) at 1:20,000, developed using Supersignal Pico (Pierce) and imaged on a Kodak imagestation 440CF. For quantification of CtrA levels in wild-type and mutant strains, four replicates of each sample were loaded on one gel and treated as described above. Once exposed, Kodak Molecular Imaging Software version 4.0.3 was used to quantify the intensity of each band and band intensities were averaged for wild-type and mutant.

#### lacZ fusions of wild-type and mutant ctrA promoters

The *ctrAP2*::Mn promoter was PCR amplified using the primers M134UP and M134DN (Table 3), incorporating EcoRI and XbaI restriction sites, respectively. The wild-type promoter was amplified using the primers M134DN and CtrAlacUp (Table 3). The digested fragments containing the promoter regions were cloned into the *lacZ* containing plasmid pLac290 [43].

#### β-Galactosidase assay

Plasmids carrying promoter fusions to *lacZ* were transferred to YB3558 and CB15 by conjugal mating. The resulting transformants were grown to an OD_600_ of 0.4 to 0.6 in liquid PYE supplemented with tetracycline. Cells were added to three tubes containing Z-buffer (60 mM sodium phosphate (dibasic), 40 mM sodium phosphate (monobasic), pH 7.0, 10 mM potassium chloride, 1 mM magnesium sulfate, 50 mM β-mercaptoethanol) to a final volume of 800 μl, and 25 μl 0.1% w/v SDS was added. To assay the LacZ activity in the samples, 200 μl of 2-nitrophenyl β-D-galactopyranoside (ONPG) (4 mg/ml in 0.1 M potassium phosphate buffer, pH 7.0) was added to each sample, and reactions were stopped by the addition of 400 μl 1 M sodium carbonate. The time between addition of ONPG and stopping the reaction was recorded. Samples were centrifuged for 5 minutes to pellet cells and debris, then the absorbance at 420 nm (A_420_) of each sample was measured and recorded. β-galactosidase activity was calculated using the formula (A_420_ × 1000)/(OD_600_ × time (min) × volume of cells used (ml)).

## Competing interests

The authors declare that they have no competing interests.

## Authors’ contributions

DK designed experiments, performed the transposon mutagenesis, mutant screening and growth curves, analyzed data, and wrote the manuscript. DK contributed to the microscopy, phage assays and swarm assay. PDC designed experiments, contributed to the microscopy, phage assays and swarm assay, analyzed data, and PDC performed the *lacZ* expression studies and wrote the manuscript. Y.V.B designed experiments, analyzed data, and wrote the manuscript. All authors have read and approved the final manuscript.
